# The role of mindful acceptance and lucid dreaming in nightmare frequency and distress

**DOI:** 10.1038/s41598-022-19624-4

**Published:** 2022-09-21

**Authors:** Sofia Tzioridou, Martin Dresler, Kristian Sandberg, Erik M. Mueller

**Affiliations:** 1 Department of Psychology, Philipps University Marburg, Marburg, Germany; 2 Donders Institute for Brain, Cognition and Behaviour, Radboud University Medical Centre, Nijmegen, The Netherlands; 3 Center of Functionally Integrative Neuroscience, Aarhus University, Aarhus, Denmark

**Keywords:** Psychology, Human behaviour

## Abstract

A theoretical and empirical association between lucid dreaming and mindfulness, as well as lucid dreaming and nightmares has previously been observed; however, the relationship between nightmares and mindfulness has received surprisingly little attention. Here, we present the findings of two studies exploring the relation of nightmare frequency and distress with two components of mindfulness, termed presence and acceptance, as well as lucid dreaming. Study 1 (N = 338) consisted of a low percentage of frequent lucid dreamers whereas Study 2 (N = 187) consisted primarily of frequent lucid dreamers that used lucid dream induction training techniques and meditation. Across studies, nightmare-related variables showed a more robust association with mindful acceptance as opposed to mindful presence. Moreover, individuals with high levels of meditation expertise and practice of lucid dreaming induction techniques reported lower nightmare frequency. Finally, in Study 2, which consisted of frequent lucid dreamers, a positive correlation between lucid dreaming frequency and mindfulness was apparent. The present findings support the notion that wakeful mindfulness is associated with the quality of dreams and extend previous research by suggesting a disentangled role of the two facets of mindfulness in dream variation. This association remains open for experimental manipulation, the result of which could have clinical implications.

## Introduction

Nightmares, defined as very disturbing dreams that awaken the sleeper (according to the International Classification of Sleep Disorders [ICSD-3] of the American Academy of Sleep Medicine (AASM)^1^, are closely associated with stress and ill-being^2,3^. On the other side, dispositional mindfulness, a concept inspired by Buddhist traditions, has been found to reduce stress^4,5^, improve sleep^6^ and promote mental well-being^7,8^. Dispositional mindfulness has been psychometrically operationalized as unidimensional or consisting of several components, which might divergently relate with well-being and ill-being related variables^9^. Lucid dreaming—i.e. the phenomenon of becoming aware of the current dream state during ongoing sleep—has been associated with alleviated nightmare distress on the one hand^10^, and increased trait mindfulness on the other^11^. Even though an indirect link between mindfulness and nightmare frequency and distress can be assumed and some findings support it^12^, more direct relationships have received little attention. Mindfulness can be trained through meditation practices and mindfulness based interventions^7,13^, which can easily be integrated into already existing clinical applications targeting nightmare disorders.

In the present paper, we aim to accentuate the role of mindfulness on dream variation by briefly reviewing the clinical aspect of nightmares and therapy approaches, such as lucid dreaming therapy, as well as the theoretical and empirical association of dispositional mindfulness and dreams. We then proceed to present our findings considering the relations among nightmares, two components of mindfulness, namely mindful presence and acceptance, and lucid dreams.

### Nightmare disorder and clinical interventions

Healthy individuals occasionally experience nightmares of various content and emotional intensity^14,15^. However, nightmare disorder is quite common, with a prevalence of around 4% in the adult population of the United States^1^. Nightmare disorder is defined based on nightmare frequency, as well as on the distress caused by the dream^1^, which is related to the affective adjustment during wake^16^.

Subsequently, nightmare disorder can result in sleep avoidance and deprivation, mood disturbance, cognitive and social function impairments, thus it can significantly reduce quality of life^17,18^. Recurrent nightmares are prominent in post-traumatic stress disorder (PTSD)^17,18^, anxiety^19–21^ and depression^22–24^ and have been associated with increased risk of suicide^20,25^ even after controlling for other risk factors^26,27^. If treated, there is a substantial improvement in sleep quality and insomnia symptoms, daytime fatigue and sleepiness are reduced^28–30^.

Taking the above into account, a variety of treatment approaches, both pharmacological and behavioral, have been suggested. Some of the behavioral and psychological treatment options include lucid dreaming therapy, image rehearsal therapy (IRT), cognitive behavioral therapy for insomnia (CBT-I), exposure, relaxation and rescripting therapy (ERRT), hypnosis, progressive deep muscle relaxation, sleep dynamic therapy, systematic desensitization etc.^18,31^.

Lucid dreaming therapy has been suggested to be beneficial as a treatment approach, mostly in combination with other behavioral treatments^18^. If trained, lucid dreaming could reduce nightmares by resignifying the dream scene, meaning reducing the negative emotions, such as fear and threat, which arise during a nightmare, by realizing it is just a dream or even by changing the oneiric storyline^32^. It is a trainable technique that could emancipate patients from their nightmares. Some studies suggest that this empowerment about one’s dreams can reduce nightmare frequency even without reaching lucidity^33^. However, conclusions about the efficacy of lucid dreaming treatment mainly rely on case reports or in some cases non-significant positive outcomes^32^ of quite underpowered empirical studies.

### Nightmares and lucid dreaming

Apart from clinical studies, surveys investigating the relationship of nightmares and lucid dreaming have been conducted. In a cross-sectional study, about 64% of a sample highly interested in lucid dreaming seem to have chosen lucid dreams in order to change nightmares or bad dreams into a more pleasant experience^34^. Moreover, narcolepsy patients, who tend to experience more lucid dreams than controls, report a positive impact of dream lucidity on distress arising from nightmares^10^. It is important to note here that in spite of the fact that nightmare frequency and nightmare distress are related, a differentiation of the two is pivotal, as a variety of factors that do not relate to nightmare frequency, can contribute to and be influenced by nightmare distress^16,35–37^. It is also suggested that frequent lucid dreamers tend to encounter less threatening figures in their dreams since they can change the plot of the dream^38,39^. However, a number of cross-sectional studies have found a positive correlation of nightmare frequency and lucid dreaming frequency^40–42^ or dream awareness^43^, which stands even after controlling for dream recall^40,41^. Since participants who report higher dream recall, also report nightmares and lucid dreams more often, it is a necessity to control for the dream recall variable^41^. The aforementioned finding of multiple studies seems to support reports of lucid dreamers that nightmares, especially recurrent nightmares, can trigger lucidity^44,45^.

### Mindfulness

Lucid dreaming might be suggested as an antidote against nightmares, but its clinical use still remains quite narrow and the findings supporting it are scarce^32^. The beneficial role of mindfulness on psychological and physical health is on the other hand much more robust in the literature^46,47^. Mindfulness, an individual disposition that can be enhanced through mindfulness meditation practice^7^, can be described as the ability to be aware of the present moment and experience it with an open and non-judging attitude^48,49^. It has been found to increase subjective well-being and reduce common forms of psychological distress^46^ and mindfulness-based therapy has been found to successfully reduce anxiety and mood problems in clinical populations^50^. Its application received increased interest in Western medical and mental health contexts some decades ago^46^ and in an attempt to better define dispositional mindfulness conceptually and operationally, multiple suggestions describing a uni-^7^, dual-^51^ or multi-^52,53^ dimensional construct have been made with most researchers following the two-component model of mindfulness proposed by Bishop et al.^46,54^. The first component, named Self-Regulation of Attention, is the ability of bringing awareness to the current experience by regulating the focus of attention. The second component, Orientation to Experience, involves adopting an orientation characterized by curiosity, openness and acceptance toward one’s experiences in the present moment^54^. This distinction of the two components seems to be important, as studies have found that the two components do not exert the same impact on well-being and ill-being^55^. The 14-item Freiburg Mindfulness Inventory (FMI)^56^, which is used in the present study, was created as a measure of a unidimensional concept of mindfulness, but it was later suggested it can be divided into two factors of mindfulness, Presence and Acceptance^9^. These two factors are in line with the model and descriptions proposed by Bishop et al.^54^. Presence, conceptually similar to Regulation of Attention described above, refers to the ability to be fully aware of internal and external experiences of the present moment, while Acceptance, conceptually corresponding to the aforementioned component named Orientation to Experience, refers to a non-judging, curious and open mindset towards these experiences^9,11,54^. Studies using this particular inventory found that Acceptance is the component that seems to influence depression and anxiety, whereas Presence exerts an impact indirectly by supporting the development of an accepting attitude^9^. Keeping the aforementioned definitions in mind, below we will present the theoretical and empirical association of mindfulness with dreams.

### Mindfulness and lucid dreaming

A great variation in consciousness is experienced constantly during the sleep–wake cycle in humans. Non-lucid dreaming is considered to consist only of primary consciousness, meaning perception and emotions, while lacking higher order (secondary) consciousness, like self-reflective awareness, volition and meta-cognition, which is present during wakefulness^57,58^. Therefore, in non-lucid dreaming, people do not realize they are dreaming. However, there are experiences in wakefulness, such as mind wandering and automatic behaviors, when higher secondary consciousness seems to be absent^59^. In addition, the rare state of lucid dreaming indicates, by definition, the presence of reflective capabilities with higher order aspects of consciousness, such as metacognition^60–62^ and volition^63^. Neuroscientific findings support this by revealing shared neural mechanisms, located in the prefrontal brain regions (BA9/10), between lucid dreaming and metacognition^62^.

Mindfulness is also related to metacognition^64–66^ and seems to influence waking states in which secondary consciousness is considered absent^67–70^. Notably, the neural correlates of dispositional mindfulness have been located in the same brain regions (medial prefrontal cortex) as metacognition and lucid dreaming^39,71^. Based on the aforementioned theoretical association, it could be hypothesized that mindfulness training could also exert an influence on dreaming via altering meta-cognition, therefore increasing the likelihood of experiencing lucid dreams.

Indeed, apart from the conjectural relation described above, lucid dreaming seems to have an empirical association with aspects of trait mindfulness^11,72^. Meditation practice has been suggested to promote lucidity in dreams^72–75^. In fact, studies report mindfulness in wakefulness to be positively related to lucidity in dreams, but only in participants who are practicing meditation^11,72^. In addition, while meditation expertise seems to be related to lucid dream frequency in some^72^ but not other^11^ studies, an 8-week mindfulness based stress reduction course did not change lucid dreaming frequency^72^.

In regard to the components of mindfulness, Presence, as measured with the FMI, was found to be associated with lucidity in dreams more robustly than Acceptance^11^. As discussed in Stumbrys et al.^11^, mindful acceptance differs from lucidity; Acceptance refers to a non-judging and accepting attitude toward emotions and experiences with no intention to change or control them^54,76^, while in dream lucidity, the dreamer is usually actively changing the dream environment and takes action to control the dream narrative according to their will^11,77^. In line with mindful presence, which by definition represents the increased ability to be aware of internal experiences, in dream lucidity the dreamer has to be aware of the mental event they experience, meaning that they are aware that they are dreaming. In another study, facets comparable to Presence here, were also found to be related to lucidity in dreams, whereas facets closer to Acceptance did not show this relation^72^.

### Mindfulness and nightmares

Despite the fact that, as seen above, an association between lucid dreaming and mindfulness, as well as lucid dreaming and nightmares^41,42^ has been observed, the relationship between nightmares and mindfulness has received surprisingly little attention, especially when considering that mindfulness interventions seem to reduce stress^4,5,78,79^, improve sleep^6,80–82^ and ameliorate sleep disturbances, that arise from stress^83^.

According to the continuity hypothesis, waking states and concerns are reflected in dream imagery^84,85^ and neuroscientific findings suggest that waking emotional patterns are preserved in dreams, influencing the dream contents. In this regard, studies have found that if someone is experiencing symptoms of anxiety or depression during waking life, this could also be evident in their dream imagery^86,87^. Moreover, it has been suggested that after only one week of meditation, anxiety and depression scores decrease and dream imagery changes^87^. Anxiety has also been linked to negative dream affect, whereas peace of mind, described as the inner peace and harmony, is related to positive dream affect^88^.

To our knowledge, two studies have investigated the relation of mindfulness and disturbed dreaming. In one study, mindfulness was found to be inversely related to disturbed dreaming and dream anxiety and to predict less severe dream disturbances after controlling for trait anxiety^12^. In the other study, mindfulness was negatively correlated with nightmare frequency^89^. However, both these studies assessed and analyzed mindfulness as a unidimensional construct. We argue here that as seen above with Presence and lucid dreaming^11^, the components of mindfulness might play different roles in relation to nightmares. Based on literature suggesting that mindful acceptance has the leading role when it comes to psychological well-being^55^, as well as influencing depression and anxiety^9^, we could assume three possible pathways of how mindful acceptance might also have a leading role in relation to nightmares. With the continuity hypothesis in mind, the first could be that mindful acceptance is helpful with anxiety and depression in waking and this is also apparent in the dream imagery. The second interpretation would be that by adopting an attitude of curiosity, openness and acceptance, one would allow oneself to experience the dream stressors as they come without the need to avoid or suppress them, eventually making them less distressing. Indeed, mindfulness has been widely viewed as an important skill facilitating emotional regulation and promoting positive emotional states^7,90,91^. Thirdly, via the same mechanism, one might experience nightmares, but instead of resorting to preoccupation or suppression of the experience when they wake up, they embrace the experience, making it less distressing and memorable. The latter is supported by evidence that mindfulness promotes quicker recovery from unpleasant emotional states^92^. To put it concisely, either the dreams are different because the waking state has changed or the dream imagery stays the same, but is interpreted or perceived as less distressing during dreaming or after waking up.

Overall, the relation of nightmares to mental ill-being has been well established^19–27^, but research on preventive factors, such as mindfulness, seems to have been widely neglected. Even though a study using the FMI has already found a negative correlation between the total FMI score and nightmare frequency^89^, the relation of the particular facets of mindfulness has not been previously discussed. As mentioned above, the two components do not seem to affect well-being and ill-being the same way^55^, leading us to the assumption that a similar observation might be apparent for dream variation as well, namely nightmares and lucid dreaming.

As discussed above, lucid dreaming has been found to be associated with both nightmares^10,40–43^ and mindfulness^11,72^, as well as with the mindfulness component presence^11^. However, studies on the topic have presented data of samples with more frequent lucid dreamers than the general population. Here, we present the findings of two questionnaire-based studies with samples that plainly differ on the engagement with lucid dreaming.

In order to extend previous findings and elucidate the association of mindfulness and dreaming, in the present paper, we examine the relationship among dream variation, more specifically nightmares and lucid dreams, and the two aspects of mindfulness, Presence and Acceptance, as measured with the 14-item FMI^56^. In one of the studies, we also investigate both nightmare frequency and distress, an important distinction when measuring nightmares as mentioned above^16,35–37^.

## Methods

Data were gathered in the context of two separate large-scale studies. Data from Study 1 were gathered in the context of EU COST Action CA18106 *The Neural Architecture of Consciousness* as part of a larger dataset from Aarhus University, Denmark. The study was approved by the local ethics committee, De Videnskabsetiske Komitéer for Region Midtjylland and all methods were performed in accordance with the relevant guidelines and regulations.

Data from Study 2 were collected via an online survey conducted by the Psychology department of Marburg University with the intention of investigating meditation techniques and lucid dreaming.

### Participants

For Study 1, 338 healthy participants (201 females) gave informed consent to participate in the survey. The mean age was 25.29 (SD = 4.94). For comparison to Study 2, the median age was 24.33 years (18–49).

For Study 2, 187 (120 females, 60 males, 7 other/NA) healthy participants gave their consent and completed an online survey. The age was measured as a categorical variable, median is 25–34 years old with 25.1%, 43.9% were older than 35 and 31% younger than 24. Participants in Study 2 were screened as to how often they recalled their dreams with the minimum acceptance criterion being three times a week.

The survey (Study 2) was advertised on international sites related to lucid dreaming as well as local survey websites and the university of Marburg recruitment platform. This resulted in a non-representative sample; participants were experienced with lucid dreaming training and have reported more lucid dreams than the usual population mean as discussed below. This allowed us to perform the analysis on two populations with different lucid dreaming frequency characteristics, experienced lucid dreamers (Study 2) and naive participants (Study 1).

### Materials and procedure

For Study 1, all participants completed the Freiburg Mindfulness Inventory (FMI) with 14 items and a 4-point rating scale^56^, a 7-point rating item developed by Schredl^93^ assessing their dream recall frequency and two 8-point rating scales developed by Stumbrys et al.^94^ measuring nightmare and lucid dream frequency^94^, all in English. All the dream related questions are also included in the Mannheim Dream Questionnaire (MADRE)^95^. The nightmare and lucid dreaming questions were accompanied with the definition of the concepts mentioned in the original questionnaires^94,95^ in order to ensure that the participants understood the concepts before replying to the question. They were presented along with 20 other psychological questionnaires in an online questionnaire session with a total duration of around 70 min. The questionnaires covered a broad range of psychological characteristics including self-reported memory, social network size, perceived stress, impulsiveness, musical sophistication and cognitive failures. Participants were instructed to complete the questionnaire session from home and to ensure that it was completed in a quiet, undisturbed setting.

For Study 2, in addition to screening and demographic data, the survey included the two scales on nightmare and lucid dream frequency mentioned above^94^, as well as the definition of the concepts^95^. It also included the FMI-14^56^, several questions assessing meditation experience, expertise, duration, type and further questions assessing sleep, chronotypes and personality. An item from the MADRE assessing nightmare distress with a five-point scale^95^ was also included in the questionnaire, but participants were given the opportunity to skip the question as “not applicable” resulting to an n = 134 for this particular item. The whole survey lasted about 10 min in total. The online questionnaire was generated using SoSci Survey^96^ and was made available to users via www.soscisurvey.de. The survey was conducted in English and was anonymous; however, participants were given the opportunity to enter a lottery in order to win one of three 20-dollar gift certificates, for which an e-mail address was required.

Both studies ensured that all questions were answered by not allowing participants to continue if there were missing responses, except for the nightmare distress question of Study 2 as mentioned above.

### Data preparation and statistical analysis

For both studies, a Total Mindfulness score was calculated from all 14 items along with a score for Acceptance (8 items) and Presence (6 items) following the division of Kohls et al.^9^. Dream data were recoded to units of mornings per week for the dream recall scale and units of frequency per month for the nightmare and lucid dream frequency items as indicated by Stumbrys et al.^94^. Meditation expertise was calculated by summing the ratings of three items, meditation session frequency (how often they meditate), meditation experience (for how long they have been meditating for) and meditation session duration.

Next, the data were examined in terms of suitability for linear analyses. As several variables did not fulfil the requirements for parametric testing (ordinal data, normal distribution etc.), correlations were assessed with the Spearman Rho test. In addition, ordinal logistic regression analysis was performed as well as the Mann–Whitney U test.

The Jamovi open statistical platform (Version 1.6.23) was used for statistical analyses^97^.

## Results

The descriptive statistics for the samples of both studies are summarized in Table 1 in order to facilitate comparison.

**Table 1:** Descriptive statistics of Study 1 and Study 2.

Variables	Study 1 (N = 338)	Study 2 (N = 187)
Dream recall per week	1 (0–6.5)/Mdn (range)	6.5 (3.5–6.5)/Mdn (range)
Nightmares per month	0.25 (0–18)/Mdn (range)	0.25 (0–18)/Mdn (range)
LD per month	0.083 (0–18)/Mdn (range)	2.5 (0–18)/Mdn (range)
FMI score	36.46 (5.98)/Mean (SD)	39 (7.37)/Mean (SD)
> 1 nightmare(s) per week	7.6%	13.9%
> 1 LD per month	29.9%	71.1%
Users of LD training techniques	13%	60.4%
Meditation	n.a	77.5%

*LD* Lucid dreaming, *FMI* Freiburg mindfulness inventory.

In Study 1, less than one-third of the participants reported having one or more lucid dreams per month, which classifies them as frequent lucid dreamers^98^. The FMI score mean was 36.46 (SD = 5.98). For comparison, the mean score of FMI for the normal sample in the study of Walach et al.^56^ was 37.24 (SD = 5.63).

In Study 2, where participants were positively selected with regard to lucid-dreaming, more than two-thirds of the participants reported having more than one lucid dream per month and more than half had already tried one or more lucid dreaming techniques, which allowed them to experience lucid dreams more frequently than the general public (24.7% as reported in Hess, Schredl and Goritz^40^). Participants of Study 2 were also engaging in different types of meditation with 77.5% reporting that they have practiced meditation at some point during their lives. The average FMI score was 39 (SD = 7.37). For comparison, the mean score of FMI for a sample with high lucid dreaming interest, was also 39 (SD = 6.3).

### Nightmare frequency

#### Study 1

Spearman correlations show that nightmare frequency was negatively correlated with the total mindfulness score as measured with the FMI, *rs*(338) = − 0.193, *p* < 0.001. The correlation of the two mindfulness facets separately; Presence: *rs*(338) = − 0.094, *p* = 0.084, Acceptance *rs*(338) = − 0.222, *p* < 0.001. Lucid dreaming frequency showed a positive correlation with nightmare frequency *rs*(338) = 0.238, *p* < 0.001. Age showed no association with the aforementioned variables.

As suggested in the literature, controlling for dream recall is a necessity as the variable is associated with both nightmare and lucid dream frequency and can influence their relationship^41^. Taking that into account, partial correlations with dream recall as a control variable were calculated (see Table 2).

**Table 2:** Spearman rho partial correlations (control variable: dream recall) of Study 1.

	1	2	3	4	5	6
1) Nightmare frequency	–					
2) FMI total	**− 0.228** ***p* < 0.001**	–				
3) FMI presence	**− 0.124** ***p* = 0.023**	**0.817** ***p* < 0.001**	–			
4) FMI acceptance	**− 0.255** ***p* < 0.001**	**0.926** ***p* < 0.001**	**0.556** ***p* < 0.001**	–		
5) LD frequency	**0.147** ***p* = 0.007**	0.047 *p* = 0.389	0.028 *p* = 0.607	0.055 *p* = 0.312	–	
6) Age	− 0.070 *p* = 0.200	0.009 *p* = 0.872	− 0.034 *p* = 0.538	0.049 *p* = 0.372	0.079 *p* = 0.146	–

*FMI* Freiburg mindfulness inventory. *LD* Lucid dreaming. N = 338.

In order to evaluate the influence of the two components of mindfulness as assessed by the FMI on nightmare frequency, we conducted an ordinal logistic regression with the two FMI subscales, Presence and Acceptance, as predictors while controlling for age, gender and dream recall.

There was a main effect of FMI Acceptance on nightmare frequency as can been seen in Table 3, but no effect of FMI Presence.

**Table 3:** Ordinal Regression for Nightmare Frequency in Study 1 and Study 2.

Variable	Study 1 (N = 338)				Study 2 (N = 187)			
	*β*	*SE*	*χ*^*2*^	*p* value	*β*	*SE*	*χ*^*2*^	*p* value
FMI acceptance	− 0.108	0.030	12.452	< 0.001	− 0.075	0.038	3.838	0.050
FMI presence	− 0.003	0.041	0.006	0.936	0.081	0.055	2.127	0.145
LD frequency	0.046	0.028	2.731	0.098	0.034	0.021	2.65	0.104

*FMI* Freiburg mindfulness inventory, *LD* Lucid dreaming. Age, gender and dream recall were included as covariates. The values reported here for FMI Presence and FMI Acceptance are before adding LD frequency in the model.

Subsequently, we included the variable lucid dream frequency in the model in order to control its effect. There was no effect of lucid dreaming frequency on nightmare frequency. The coefficients of FMI Acceptance remained unchanged.

#### Study 2

The same analyses was performed for Study 2. The Spearman correlations before controlling for dream recall showed a non-significant negative correlation for nightmare frequency and FMI total *rs*(187) = − 0.112, *p* = 0.126, as well as the two mindfulness components, FMI Presence *rs*(187) = − 0.056, *p* = 0.449 and FMI Acceptance *rs*(187) = − 0.140, *p* = 0.055. FMI Acceptance was negatively correlated with nightmare frequency when controlling for dream recall (see Table 4). No association was observed between nightmare frequency and lucid dream frequency.

**Table 4:** Spearman rho partial correlations (control variable: dream recall) of Study 2.

	1	2	3	4	5	6	7
1) Nightmare frequency	–						
2) Nightmare distress (n = 134)	**0.229** ***p* = 0.008**	–					
3) FMI total	− 0.123 *p* = 0.094	− 0.154 *p* = 0.077	–				
4) FMI presence	− 0.064 *p* = 0.389	− 0.088 *p* = 0.313	0.**869** ** < 0.001**	–			
5) FMI acceptance	**− 0.153** ***p* = 0.037**	− 0.164 *p* = 0.059	**0.929** ***p* < 0.001**	**0.641** ** < 0.001**	–		
6) LD frequency	**0**.024 *p* = 0.740	**− 0.201** ***p* = 0.021**	**0.255** ***p* < 0.001**	**0.229** ***p* = 0.002**	**0.235** ***p* = 0.001**	–	
7) Age	**− 0.332** ***p* < 0.001**	− 0.095 *p* = 0.277	**0.285** ***p* < 0.001**	**0.249** ***p* < 0.001**	**0.250** ** < 0.001**	0.138 *p* = 0.060	–

*FMI* Freiburg mindfulness inventory, *LD* Lucid dreaming. N = 187.

In agreement with Study 1, when performing an ordinal regression, there was only a main effect of FMI Acceptance on nightmare frequency when controlling for age, gender and dream recall. The results are summarized in Table 3.

The effect of lucid dream frequency was non-significant and the effect of FMI Acceptance remained after the addition of lucid dream frequency in the model, *β* = − 0.084, *SE* = 0.039, *χ*^*2*^(1, N = 187) = 4.77, *OR* = 0.91, *p* = 0.029.

### Nightmare distress

Nightmare distress was negatively correlated with mindfulness and its components, but only the association with FMI Acceptance was significant, *rs*(134) = − 0.182, *p* = 0.035. FMI Presence *rs*(134) = − 0.099, *p* = 0.257 and FMI total *rs*(134) = − 0.169, *p* < 0.051. Furthermore, a bivariate, *rs*(134) = − 0.193, *p* = 0.026 and a partial correlation (see Table 4) showed a negative association between nightmare distress and lucid dream frequency.

When testing the effects of the mindfulness components with age and gender as covariates, only FMI Acceptance significantly predicted nightmare distress, After controlling for lucid dream frequency, FMI Acceptance did not predict nightmare distress, *β* = − 0.081, *SE* = 0.045, *χ*^2^(1, N = 134) = 3.25, *OR* = 0.92, *p* = 0.071 and the effect of lucid dream frequency on nightmare distress was not significant (see Table 5).

**Table 5:** Ordinal Regression for Nightmare Distress in Study 2.

Variable	*β*	*SE*	*χ*^*2*^	*p value*
FMI acceptance	− 0.089	0.045	3.943	0.047
FMI presence	0.017	0.064	0.072	0.788
LD frequency	− 0.036	0.024	2.223	0.136

*FMI* Freiburg mindfulness inventory, *LD* Lucid dreaming. Age and gender were included as covariates. The values reported here for FMI Presence and Acceptance are before adding LD frequency in the model. n = 134.

### Lucid dreaming

#### Study 1

Lucid dream frequency did not correlate with mindfulness and its components in Study 1 with both bivariate and partial correlations producing very weak, non-significant correlations (see Table 2 for partial correlation). An ordinal regression on lucid dream frequency with the two FMI components as predictors while controlling for dream recall, age and gender was conducted. There was no significant effect of the variables on lucid dream frequency. However, exploratory correlations revealed a negative correlation between nightmare frequency and an item assessing engagement in lucid dreaming techniques after controlling for dream recall, *rs*(338) = − 0.143, *p* = 0.009 (bivariate correlation: *rs*(338) = − 0.103, *p* = 0.057).

#### Study 2

Lucid dreaming was positively correlated with the FMI total score, *rs*(187) = 0.265, *p* < 0.001, FMI Presence, *rs*(187) = 0.238, *p* = 0.001 and FMI Acceptance, *rs*(187) = 0.246, *p* < 0.001 (see Table 4 for partial correlation).

None of the two components of mindfulness significantly predicted lucid dreaming when conducting an ordinal regression as mentioned above.

Similar to Study 1, however, the more lucid dream induction techniques participants reported to have used, the less frequent *rs*(187) = − 0.267, *p* < 0.001 and distressing *rs*(134) = − 0.256, *p* = 0.003 they reported their nightmares to be. The coefficients did not change substantially when controlled for dream recall.

### Meditation

In study 2, where meditation experience was also examined, a Mann–Whitney test indicated that the participants who have practiced meditation reported significantly lower nightmare frequency (*Mdn* = 0.25) than the participants who reported that they have never practiced meditation, *Mdn* = 1, *U* (N_mediation_ = 145, N_no-meditation_ = 42) = 2050, *p* = 0.001, *r*_*rb*_ = 0.33. There was no difference between the two meditation groups (Yes/No) when it came to nightmare distress.

Meditation expertise was negatively correlated with nightmare frequency, *rs*(187) = − 0.322, *p* < 0.001, but not nightmare distress, *rs*(134) = − 0.052, *p* = 0.55. The coefficients did not change substantially when controlled for dream recall.

In order to assess whether the relaxation effect of meditation alone influences the relationship of mindfulness and nightmares, an ordinal logistic regression with an interaction term was calculated with the aim to measure whether meditation expertise moderates the effect of FMI Acceptance on nightmare frequency, as well as nightmare distress while controlling for age, gender and dream recall. There was no effect of the FMI Acceptance on nightmare frequency *β* = − 0.032, *SE* = 0.028, *χ*^2^(1, N = 187) = 1.24, *OR* = 0.96, *p* = 0.266 when meditation expertise was included in the model, but no moderation occurred. The effect of meditation expertise on nightmare frequency was significant, *β* = − 0.084, *SE* = 0.030, *χ*^2^(1, N = 187) = 7.66, *OR* = 0.92, *p* = 0.006. On the other hand, only FMI Acceptance significantly predicted nightmare distress *β* = − 0.076, *SE* = 0.034, *χ*^2^(1, N = 134) = 4.86, *OR* = 0.93, *p* = 0.027 with no effect of meditation expertise *β* = 4.14e−4, *SE* = 0.034, *χ*^2^(1, N = 134) = 1.44e−4, *OR* = 1, *p* = 0.990.

A partial Spearman correlation (control: dream recall) revealed a positive correlation between nightmare frequency and the last time meditation was performed (*rs*(145) = 0.175, *p* = 0.036), meaning the longer it has been since the last meditation session, the higher the nightmare frequency. Moreover, A Kruskal–Wallis test showed that the meditation category (focused attention, open monitoring or combined) did not significantly affect nightmare frequency, *H*(2, N = 140) = 2.12 , *p* = 0.346. Finally, the number of lucid dreaming techniques used was positively correlated with meditation expertise, (*rs*(187) = 0.405, *p* < 0.001).

## Discussion

Mindfulness is negatively associated with nightmare frequency and our results indicate mindful acceptance as the main component related to nightmare frequency, even after we controlled for lucid dreaming frequency. Acceptance, as measured by the FMI, also seems to be associated with nightmare distress. A positive correlation between mindfulness and lucid dream frequency was prominent in Study 2, as found in other studies, but we did not find an association of a particular mindfulness component with lucid dream frequency. The use of lucid dream induction techniques seems to be associated with nightmare frequency and distress, a relation that will be discussed in more detail below.

Meditation practice plays a role, as results showed that participants who reported having practiced meditation at some point in their lives, also reported lower nightmare frequency. However, meditation expertise did not seem to moderate the relationship of nightmare frequency and Acceptance. The specific type of meditation practiced was not found to play a role in relation to nightmare frequency, however, the time passed since the last meditation session is positively related to nightmare frequency. Overall, the present findings support the idea that wakeful mindfulness is associated with the quality of dreams and more specifically that facets of mindfulness might have separate roles in dream variation.

Based on the results of the two studies, nightmare frequency was negatively correlated with mindfulness, more robustly with the facet of Acceptance as measured with the FMI, extending the previous findings on a unidimensional measure of mindfulness and dream disturbances^12,89^. In fact, Acceptance explained nightmare frequency in both studies and explained nightmare distress measured in Study 2. It should be noted, that partial correlations between nightmare distress and the mindfulness components depicted in Table 4 should be cautiously interpreted, if at all, as controlling for dream recall here was kept as a matter of consistency rather than necessity. Overall, FMI Acceptance seems to be associated with nightmare frequency and distress in a more robust manner than FMI Presence, which is in congruence with literature suggesting that mindful acceptance is the main feature of mindfulness that both reduces distress and promotes psychological well-being^51,55^. Additionally, previous findings showed that attention monitoring, similar to mindful presence here, hardly predicted ill-being while most benefits on psychological well-being and ill-being depend on mindful acceptance alone^55^.

In more detail, mindful acceptance alone has been associated with lower stress, depression and anxiety^99^ and lower post-traumatic stress symptoms^100,101^, all of which have been associated with higher nightmare experiences^24,102,103^. It has been suggested that stress is mediating the positive relationship between mindfulness and sleep quality and well-being^83^, which makes it highly possible that a similar mediation is taking place in the relationship we observed here, between mindfulness and nightmares. It is therefore of high importance that future studies investigate this possibility.

To our knowledge, a relationship between mindful acceptance and nightmares has not been previously described in the literature. This lack of evidence on how mindfulness could potentially benefit the treatment of nightmares might explain why mindfulness training has not yet received a more prominent position as a complementary method of the treatment approaches of nightmare disorder. Our findings support the theoretical assumptions of how mindful acceptance could influence nightmares, which were presented earlier. Future studies could particularly investigate the likelihood of each of these interpretations.

Lucid dreaming frequency was correlated with mindfulness and its components in Study 2 where the sample was constituted of a high percentage of frequent lucid dreamers (71.1%), higher than the sample from Stumbrys et al. (49.8%)^11^, where participants with high interest and/or engagement in lucid dreaming were recruited, and notably higher when compared to the sample of Study 1 (29.9%) that did not aim for a sample with interest in lucid dreaming. For comparison, other studies with “naïve” samples, reported lucid dreaming frequencies similar to Study 1 (24.7%^40^ and 36.9%^41^). However, the finding of Stumbrys et al.^11^, where FMI Presence predicted lucid dream frequency in a regression model could not be replicated in our studies. One possible explanation could be the difference between the sample characteristics in respect to meditation experience and expertise. In Study 2 here, 77.5% reported having some meditation experience with a median of 2–4 years as measured by a categorical variable, whereas in Stumbrys et al.^11^ only 22.3% of the sample reported meditation experience with a median of 3 years. It is also worth noting that the two studies also differed in respect to nationality and language characteristics of the sample as the study of Stumbrys et al.^11^ included only German-speaking participants.

Moreover, lucid dreaming frequency was negatively correlated with nightmare distress, which is in line with previous literature^10^, as discussed before. However, lucid dreaming frequency showed a positive correlation with nightmare frequency, which has been previously reported^40,41^, as nightmares, especially recurrent ones, can trigger lucidity^44^. It is worth noticing that the relation was only apparent in Study 1, in which only 10.9% of the frequent lucid dreamers had actively engaged with lucid dreaming training techniques in comparison to 67.7% of Study 2. Interestingly, other studies that show a positive correlation between lucid dream and nightmare frequency have also recruited convenience samples unrelated to lucid dreaming^40,41^, similar to Study 1. Participants with spontaneous lucid dreams might lack the empowering effect introduced by lucid dreaming training techniques that predominantly enlighten on the efficacy of controlling ones dreams. Moreover, the more lucid dreaming induction techniques our participants reported to have used, the less frequent and distressing they reported their nightmares to be. These findings support the idea, we discussed before; the empowerment someone can gain by the knowledge of potentially controlling their dreams can reduce nightmare frequency and intensity, even without reaching lucidity^32,33^. However, since the number of reported lucid dreaming training techniques is positively correlated with meditation expertise, we cannot exclude a possible additive effect of the two practices on nightmare related variables.

Meditation expertise was associated with nightmare frequency; however we found no link between meditation and nightmare distress. Meditation expertise did not moderate the relationship between mindfulness and the aforementioned variables, but the main effect of FMI Acceptance on nightmare frequency was decreased after meditation was added to the model, whereas it was increased for the nightmare distress variable. This could suggest that the relaxation effects of the meditation practice have a direct effect on nightmare frequency, possibly due to its stress-relieving properties, whereas the effect on nightmare distress might primarily be achieved through meditation practices that promote mindfulness (mindfulness based meditation). This is also supported by the positive relation of the time one abstains from practicing meditation and the increment of nightmare frequency. Moreover, the types of meditation, categorized as focused attention, open monitoring or combined, did not differ on their effect on nightmare frequency; nevertheless, these types of meditation have been found to mainly promote mindfulness^104^. Future studies should address this question and investigate how the different types of meditative practices affect dream variation and intensity.

Taking the aforementioned findings into account, mindfulness based meditation could potentially be a worthy complementary method in reducing nightmare frequency and distress in both clinical and non-clinical populations. In fact, theories that place nightmare-prone individuals within the differential susceptibility framework support the notion that nightmare sufferers may benefit from emotion regulation strategies, such as mindfulness training, due to the possibility that intense emotions, both positive and negative, may be maladaptive and induce awakenings^105^. This, in addition to the potential relationship of mindfulness and lucid dreaming, which, as discussed, is already suggested as complementary treatment for nightmare disorder, may make mindfulness a great aid for the therapeutic process and/or prevention.

While the present findings endorse the idea of different facets of mindfulness having distinct roles when it comes to their relationship to nightmares or, if combined with previous literature, with dream variation in general, our results should only be taken into account through the prism of the following limitations.

The main limitation is the cross-sectional nature of our studies that precludes causal inferences*.* As in all entirely questionnaire-based studies, correlations between constructs could in principle be driven by common method variance. Future work, perhaps with physiological measurements during sleep, could address the issue. Nevertheless, our pattern of findings cannot be explained by a global response style, which would likely have influenced the correlations of all the mindfulness subscales rather than specific correlation patterns between facets of mindfulness and dream variables. The congruence with findings of other studies also argues against a strong influence of common method variance.

Moreover, the data we analyzed here were part of bigger surveys that were not built in order to solely investigate the relationships we presented here. As mentioned, the two surveys were separate and conducted by two universities in different countries. In spite of the fact that they were both conducted in English and Study 2 was advertised in international websites, some cultural differences are expected.

Regarding the percentage of participants that experience nightmares once or more times per week, which could indicate elevated nightmare frequency, our studies did not substantially differ to each other (Study 1: 7.6%; Study 2: 13.9%) or other studies (15.8%, 10.11% as reported in Schredl and Göritz^15^ and Schredl et al.^106^ respectively). It is important to note that these studies are only constituted of samples without a particular interest or engagement to lucid dreaming, unlike Study 2. In any case, even though the findings of the two studies presented here are congruent regarding the relationship of Acceptance and nightmares, we think that the two studies should be compared with caution and critical view. The two samples allowed observations about both more experienced lucid dreamers and meditators (Study 2), as well as relatively naive participants (Study 1), to be made, but they were also differing in dream recall and possibly meditation practice and expertise, which can influence the rest of the interrelated variables. Concerning meditation, we were unable to test for differences, as Study 1 did not include questions about meditation. Furthermore, participants in Study 2 were selected based on their dream recall frequency and were recruited mostly from websites related to lucid dreaming, which may diminish the generalizability of the findings.

Finally, mindfulness is a complex construct and various questionnaires and studies conceptualize it as both unidimensional^7^ and multidimensional^52,53^. The FMI is an inventory that originally approached mindfulness as a unidimensional construct^56^ but a two factor approach is suggested for the shorter 14-item scale^9^, which was used here. Other mindfulness scales have been developed to measure up to five facets of mindfulness^52,53^. Future research should take that into account and investigate how the different suggested mindfulness components interact with dreaming experiences.

Experimental studies with naïve participants and behavioral interventions will not only advance our understanding about how mindfulness and its components are related to dream quality, but may also support the implementation of the so far neglected mindfulness-based therapy as a complementary technique to existing nightmare disorder treatments. As mentioned before, frequent nightmares can lead to sleep resistance and can have a severe effect on waking life, substantially reducing quality of life and overall well-being^17,18,21^. Nevertheless, people seem to be quite reluctant to seek professional help for their nightmares, possibly because they perceive them as “normal”^107^ since in some cases they have been experiencing nightmares since childhood^108^ or they do not perceive them as a problem worth seeking professional help for. In this regard, mindfulness and acceptance-based self-help interventions might be proven a very effective option for reducing nightmare frequency and distress, as there are evidence that such interventions help with other pathological conditions like depression and anxiety^109,110^. Moreover, a stepped care approach integrating mindfulness based prevention and intervention techniques for nightmare prone individuals is likely to appear as a more attractive and accessible method of getting help.

## Conclusions

The findings of the two studies presented here support the notion of a negative association between mindfulness and nightmares and extend previous results by suggesting mindful acceptance as the main component related to nightmare frequency and distress. Furthermore, practicing meditation and/or lucid dreaming induction techniques appears to be inversely related to nightmare frequency and in the latter case also nightmare distress. Longitudinal experimental studies further investigating the aforementioned findings would be meaningful in order to understand the complexity of dispositional mindfulness and its association to dream variation, as well as inaugurate causal relationships, which can contribute to the refinement of prevention and intervention techniques used in the treatment of nightmare disorder.

## Data Availability

The datasets generated and/or analyzed during the current study are available from the corresponding author on reasonable request.
